# Evaluation of Residual Infectivity after SARS-CoV-2 Aerosol Transmission in a Controlled Laboratory Setting

**DOI:** 10.3390/ijerph182111172

**Published:** 2021-10-24

**Authors:** Luisa Zupin, Sabina Licen, Margherita Milani, Libera Clemente, Lorenzo Martello, Sabrina Semeraro, Francesco Fontana, Maurizio Ruscio, Alessandro Miani, Sergio Crovella, Pierluigi Barbieri

**Affiliations:** 1 Institute for Maternal and Child Health, IRCCS Burlo Garofolo, Via dell’Istria 65/1, 34137 Trieste, Italy; 2 Department of Chemical and Pharmaceutical Sciences, University of Trieste, Via L. Giorgieri 1, 34127 Trieste, Italy; slicen@units.it (S.L.); lorenzo.martello@studenti.units.it (L.M.); barbierp@units.it (P.B.); 3 Department of Medicine, Surgery and Health Sciences, University of Trieste, Strada di Fiume 447, 34137 Trieste, Italy; margherita.milani@studenti.units.it; 4 Ospedale San Polo, Azienda Sanitaria Universitaria Giuliano Isontina, Via Luigi Galvani 1, 34074 Monfalcone, Italy; libera.clemente@asugi.sanita.fvg.it (L.C.); francesco.fontana@asugi.sanita.fvg.it (F.F.); 5 INSTM National Interuniversity Consortium of Materials Science and Technology, Via G. Giusti 9, 50121 Firenze, Italy; ssemeraro@units.it; 6 Ospedale Maggiore, Azienda Sanitaria Universitaria Giuliano Isontina, Piazza dell’Ospitale 1, 34129 Trieste, Italy; maurizio.ruscio@asugi.sanita.fvg.it; 7 Department of Environmental Science and Policy, University of Milan, Via Festa del Perdono 7, 20122 Milano, Italy; alessandro.miani@unimi.it; 8 Department of Biological and Environmental Sciences, College of Arts and Sciences, University of Qatar, Doha 2713, Qatar; sgrovella@qu.edu.qa

**Keywords:** SARS-CoV-2, aerosol, residual infectivity

## Abstract

Severe acute respiratory syndrome coronavirus type 2 (SARS-CoV-2) is mainly transmitted through respiratory droplets, aerosols, or direct contact with fomites from an infected subject. It has been reported that SARS-CoV-2 is stable and viable in aerosol up to 16 h in controlled laboratory conditions. However, the aerosolization conditions varied a lot between the studies. In this work, an experimental laboratory model of SARS-CoV-2 aerosolization was established, employing an impinger nebulizer, a cylindrical chamber for aerosol travel, and a SKC biosampler for the collection of particles. The efficiency of the system was assessed based on the molecular determination of the viral load in the nebulizer after the aerosolization and in the aerosol collected at the end of the travel. Moreover, the residual infectivity was tested in vitro on the Vero E6 cell line, through the observation of the cytopathic effect (CPE), and the quantification of the viral load in the supernatants at 7 days post inoculation (dpi). A high RNA viral load was found in the SKC biosampler after aerosolization, indicating that it was possible to transport a high virus titer through the 30-cm chamber with all the dilutions (initial 10^5^, 10^4^, 10^3^ plaque forming unit—PFU/mL). At the 7 dpi, an increment of the RNA viral load was determined for the dilutions 10^5^ and 10^4^ PFU/mL tested, while only the initial 10^5^ PFU/mL resulted in visible CPE. Our findings allowed us to achieve the resilience of SARS-CoV-2 in aerosol form, at a concentration comparable to those reported for clinical samples. This mode of transmission should be considered for the mitigation and preventive measures to counteract SARS-CoV-2 spreading.

## 1. Introduction

Severe acute respiratory syndrome coronavirus type 2 (SARS-CoV-2) is the etiological agent of the acute respiratory COronaVIrus Disease 2019 (COVID-19), predominantly transmitted through respiratory droplets, aerosols, or direct contact with fomites derived from an infected individual [1]. In May 2021, the American Center for Disease Control and Prevention (CDC) pointed at inhalation of very fine respiratory droplets and aerosol particles as the first of the ways of exposure to SARS-CoV-2 [2]. The relevance of aerosol transmission for COVID-19 is supported by multiple lines of evidence [3], even if issues related to the airborne transmissions are still a matter of debate [4,5] due to the high variability of size distribution of virus laden particles emitted by infected individuals [6,7], and complex dynamics and interactions among them and environmental conditions. Several factors have been reported as able to affect aerosol infectivity, such as temperature (T), relative humidity (RH), atmospheric particulate matter (PM) and oxidants as atmospheric reactive oxygen species (ROS), and solar UV rays [4,5,6,7,8,9,10,11]. Moreover, the lack of standardization in viruses’ environmental sampling [12], in atmospheric sample storage and handling for infectivity assessment should be mentioned as factors potentially limiting advancement in understanding SARS-CoV-2 environmental transmission. Reviews on environmental monitoring of viral presence have been proposed by the UK Scientific Advisory Group for Emergencies, US CDC [13,14], as well as by Rahmani et al. [15], who specifically considered SARS-CoV-2, while Borges et al. focused on reviewing indoor sampling and analysis [16]. No univocal approach has been identified as stated universally preferable. Robotto et al. [17] have proposed a preliminary strategy to SARS-CoV-2 indoor and outdoor air sampling with complementary methods in order to overcome the lack of standardization of specific devices and procedures.

The high transmissibility of SARS-CoV-2 can be ascribed to its mode of infection and molecular characteristics; indeed, the SARS-CoV-2 viral load in the naso-oropharyngeal region peaks during the first week of infection, resulting in a high risk of viral shedding and transmission through speech, cough, or sneeze at the beginning of the infection or also in the asymptomatic phase [18].

The amount of RNA virus copies emitted depends on the mode of exhalation—namely breathing, talking, sneezing, or coughing—and on the SARS-CoV-2 viral load in the mouth and nose, variable during the course of the disease [19,20,21,22]: virus genomic RNA concentration has been evaluated at up to 10^8^ viral copies mL^−1^ in asymptomatic patient and 10^11^ viral copies mL^−1^ in symptomatic ones [6].

Originally, in 1934, Wells et al. proposed a 5-µm cut-off to discriminate between the large particles with size > 5 µm, the droplets, that for their conformation are subjected to fast deposition, and the small particles, <5 µm, the aerosol, that can persist and proceed in the air for a considerable length (several minutes and meters away [23]); the same definition was recently adopted in reports by the World Health Organization (WHO) [24].

Nevertheless, aerosol scientists have provided evidence that in a real condition, a continuum of particles from 0.6 to 1000 µm containing virus can be emitted in expiratory events, and the particle sizing around 5 µm can also persist suspended in the air. Finally, large particles can also rapidly evaporate forming smaller droplet nuclei with the characteristics of aerosol [4]. A new cut-off size of 100 µm has been set as dimension above which droplets fall on surfaces within 2 m from infected emitting subjects [25], highlighting the relevance of aerosol transmission. The issue of coagulation of droplet nuclei with preexisting air particulate matter, with possible stabilization of virus, is still unclear with controversial findings [26,27,28,29], requiring further experimental studies. Both droplet and aerosol particles are generated from the lining fluid covering the airways; large particles originate from the liquid mucus–air surface instability in the upper airways, resulting in fluid wave during airflow that can be exhaled during coughing and sneezing [7], while small particles derive from the terminal bronchioles during the closing and opening of terminal airways in the tidal breathing [30].

The SARS-CoV-2 modes of transmission may be slightly different for large particles, via direct/indirect contact through mouth, nose, or conjunctiva and for small particles, through inhalation and deposition in the lower airways. Of note, also in this last scenario, a gradient of deposition of the particles in the airways should be present, from the larger that deposit in the upper airways to the smaller reaching the lower airways. Interestingly, the probability that the virus in particles smaller than 1 µm can directly reach the lower airways and establish there the site of primary replication leading to pneumonia may be very low. On the other hand, the fraction of viruses that can be deposited in extrathoracic airways through 2–3 µm particles is about 62% of the total; therefore, it can be speculated that the site of primary replication should be established in the upper airway, and from this site, the virus can reach the lung [31]. An interesting anatomic issue arises from the peculiar features of droplets generation: young children present few alveoli and bronchioles, low velocity in the exhaled air, and reduced collapse of the airways, traits that evolve and increase with age beyond adolescence [32,33]. Therefore, they should be less efficient transmitters with respect to adults [34].

There are several investigations regarding the presence of SARS-CoV-2 genomic traces in indoor and outdoor environments [35,36]; however, the methodological and instrumental standardization is lacking [16]: impactors, filters, impingers, cyclones, and recently growth tube collectors—allowing to sample efficiently bioaerosol particles with size smaller than 300 nm—have been proposed. Maximal experimental virus concentrations measured in healthcare indoor air samples have been detected in the order of 10^3^ genome viral copies per m^3^ of air [37,38], while in silico models point at possible viral concentrations until 10^6^ copies/m^3^ within indoor spaces [33,39].

Since viral RNA presence in an environmental matrix does not imply virus replication and infectivity on target biological systems, specific studies are required; at present, only a small number of researchers have succeeded in the detection of infectiousness of environmental samples [38]: two studies were conducted by collecting air from hospital units hosting COVID-19 patients [40,41] and one in a car driven by a COVID-19 positive individual [42].

Moving in the field of experimental model studies, setting an effective SARS-CoV-2 aerosolization, and controlling particle size distribution and number, aerosol transfer, and SARS-CoV-2 sampling while keeping viral infectivity are not trivial tasks and again, few works are reported in literature describing the fate of the virus after aerosolization in laboratory-controlled conditions. Aiming at defining viral persistence in air, after laboratory-controlled virus aerosolization, SARS-CoV-2 was found as infectious at 90 min [43], 3 h [44], or 16 h [45], depicting a range of variability between the studies. Moreover, the aerosolization and collection procedures were not described in detail by Fears et al. [45] and van Doreleman et al. [44], probably due to the main aims of the two works of describing the virus stability, but not reporting the technical procedures used. Smither et al. [43] were more accurate in the illustration of the experimental configuration, detailing aerosol generation, drum filling, sampling, and operative conditions, focused on airborne persistence of SARS-CoV-2 England-2 variant (GSAID Accession ID EPI_ISL_407073).

The study of conditions that allow to detect SARS-CoV-2 residual infectivity after aerosolization, air transfer, and sampling is still a relevant research and public health issue for the definition and limitation of settings posing effective risk for COVID-19 airborne transmission. The control and safety of healthcare facilities are obviously places of high concern for the overt presence of sensible individuals. In particular, ventilation of indoor spaces hosting COVID-19 positive individuals is mandatory; nevertheless, ventilation system can also transport the virus creating cross-contaminations. Adequate design and management of Heating, Ventilation and Air Conditioning (HVAC) systems—also for low-risk wards different from Intensive Care Units—represent an important non-pharmacological strategy for obtaining satisfactory indoor microbiological quality [46]. Effective bio-aerosol sampling and analysis protocols need to be implemented so to check and monitor the capacity of ventilation system in limiting the SARS-CoV-2 presence at low levels, for the safety of healthcare staff and patients.

Aiming at providing a simple setup for laboratory studies on the behavior of infectious SARS-CoV-2 in aerosol phase, we propose an experimental model for the generation and collection of bioaerosol carrying SARS-CoV-2, useful for assessing the effects of viral dilution on residual infectivity of aerosol samples, evidenced on cellular models as a cytopathic effect on Vero E6 cells, and thus on airborne transmission. Environmental viral low concentrations can occur either for weakening of the emitting viral source, i.e., when an infected individual is not in the peak of virus shedding, or for dilution due to the distance between the infected individual and the susceptible one. Moreover, previous published laboratory studies dealing with SARS-CoV-2 aerosolization [43,44,45] have used Goldberg drums as aerosol chambers, generating static suspensions with the aim of assessing viral persistency in air; here, the presented experimental model for the generation and collection of bioaerosol can also premise further development of methods for assessing Viral Filtration Efficiency [47], for testing individual protection devices, air disinfection technologies, and air samplers by using SARS-CoV-2 as challenge microorganism and not bacteria—as it is foreseen in the technical norms as EN 14683:2019 or ASTM F2101-14 Standard Test Method for Evaluating the Bacterial Filtration Efficiency (BFE) of Medical Face Mask Materials—nor bacteriophages [48] or other viruses [49].

This appears to be the first study reporting data on residual infectivity for different concentrations of SARS-CoV-2 after aerosolization by high performance Blaustein Atomizing Modules (BLAM) atomizer and optimized impinger sampling in flow conditions.

## 2. Materials and Methods

### 2.1. SARS-CoV-2 Suspension Preparation

SARS-CoV-2, previously isolated in the BSL3 facility of the San Polo hospital (ASUGI, Monfalcone, GO, Italy) from the supernatant of infected Vero E6 cell line (epithelial kidney normal cell line from *Cercopithecus aethiops*, ATCC CRL-1586), was employed in the aerosolization (Supplementary Information File S1) [50,51]. The virus was quantified by using a standard plaque forming unit (PFU) assay [52].

For aerosol generation, the virus was diluted at 10^5^, 10^4^, and 10^3^ PFU/mL in infection medium (MEM + 2% fetal bovine serum, 2 mM glutamine, and 100 U/mL penicillin/streptomycin; Euroclone, Pero, Italy).

### 2.2. The Bio-Aerosol Measuring Train

**Figure 1:**
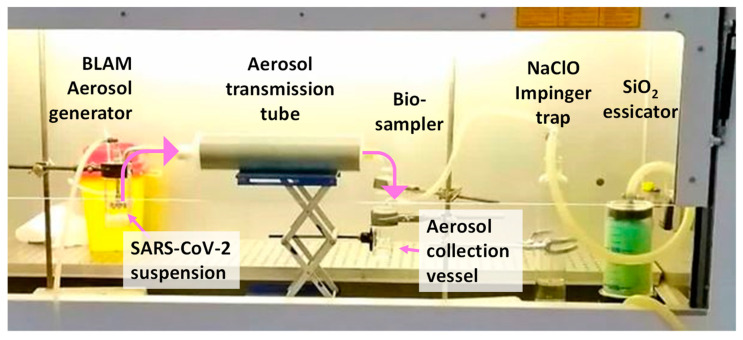
Bio-aerosol generation transmission and sampling train: The *BLAM Aerosol generator* hosts the *SARS-CoV-2 suspension*, receives an air flow from the AERO nebulizer pump (out of the figure), generates the bio-aerosol that is transmitted through the *Aerosol transmission tube,* and it is collected in the vessel of the *Biosampler*. The air flow is disinfected by passing into a *NaClO impinger trap*, and dried by a *SiO_2_ dessicator*, before entering a BioBravo pump (out of the figure) that sustains the sampling. Pink arrows show the path followed by SARS-CoV-2 aerosol.

Experiments on residual infectivity after virus aerosolization were conducted in a Celbio Model Jupiter 6 biosafety cabinet class II within the BLS3 facility of the San Polo hospital (ASUGI, Monfalcone, GO, Italy), in a sealed experimental bio-aerosol measuring train assembled as it follows. A BLAM aerosol generator (CH Technologies Inc., Westwood, NJ, USA) hosting the SARS-CoV-2 suspension in its precious jar, received an air flow—produced by AERO Particle Nebulizer pump (TCR Tecora Srl—Cogliate, MB, Italy) which is positioned outside the cabinet—generating the bio-aerosol. The BLAM aerosol generator has a filtered inlet (0.2 μm) allowing entrance of air needed to sustain the air flow in the measuring train, as detailed in the next paragraph. The aerosol is transferred into a 300-mm long and 75 mm of diameter PP cylindrical aerosol transmission tube, and from it, to a Biosampler (SKC Inc., Eighty Four, PA, USA), collecting the aerosolized SARS-CoV-2 into a collection vessel. The virus particle collection into the Biosampler is sustained by an aspiration flow of 12.5 L per minute, generated by a Bio-Bravo sampling pump (TCR Tecora Srl, Cogliate, MB, Italy). A glass impinger with sodium hypochlorite working as safety trap deactivating eventual unsampled pathogens, and a dryer filled with silica protecting the pump from vapors are posed online after the Biosampler and before the Bio-Bravo. All connections were prepared in nylon with o-rings by the mechanical workshop of the Dept. of Chemical and Pharmaceutical Sciences of the University of Trieste. The bio-aerosol measuring train has been preliminary set up and tested with *E. coli* BL21-DE3. An image of the bio-aerosol measuring train is shown in Figure 1. A detailed scheme of the bio-aerosol measuring train and images of the aerosol generator and the Biosampler are reported in the Supplementary Materials, respectively as Supplementary Figures S1–S3.

After each experiment, the set-up was carefully washed; moreover, the experimentations started from the less concentrated virus to avoid any potential residual unintentional carry-over. 

### 2.3. Aerosol Generation

Among bioaerosol generators, the Blaustein Atomizing Modules (BLAM) nebulizer (CH Technologies Inc., USA) with 8 jets has been selected, in consideration of superior nebulization efficiency [53]—as tested on *Escherichia coli*—in comparison to Sparging Liquid Aerosol Generator (SLAG) and Collision nebulizers. The BLAM does not provide impacts of bioaerosol on hard surfaces, and it requires lower pressures than the Collision nebulizer, with less stress to the microorganisms, preserving efficiently their infectivity. The virus suspension (about 5 mL) was thus added in the precious liquid well of the glass jar of an 8-jet BLAM nebulizer (CH Technologies Inc., USA) with horizontal discharge, operated in Multi Pass Atomization mode, at a flow of 8 L per minute. The aerosol was released in a 300-mm cylindrical tube with a diameter of 75 mm, subject to aspiration of the Biosampler (12.5 L per minute); aerosolization runs lasted for 5 or 10 min. Filtered air required for equilibrating flows entered into the system through a 0.2-micron Polyethersulfone (PES) filter fitted on the BLAM cap. At the end of the procedure, the remaining liquid was collected in a falcon tube and the chamber was washed with water; consecutively, a new viral suspension was added.

### 2.4. Size Distribution Temperature and Relative Humidity Assessment

The size distribution of aerosol particles generated from the infection medium (MEM + 2% fetal bovine serum, 2 mM glutamine, and 100 U/mL penicillin/streptomycin) by the 8-jet BLAM nebulizer operated at 8 L per minute, was measured in subsequent runs at the inlet and at the outlet of the cylindrical aerosol transmission tube with aspiration forced by the TCR Tecora Bio Bravo, in order to check size distribution an eventual phenomena of growth or coagulation or particle loss occurring in the aerosol tube could be evidenced. Size distribution was measured by an Optical Particle Counter GRIMM EDM 107 counting particles in the range 0.25–32 µm, with acquisition time of 6 s per single lecture.

Temperature and relative humidity (RH) were measured by using the Inkbird IBS-TH1 Plus Bluetooth thermo-hygrometer, positioned in the cylindrical chamber during aerosolization.

### 2.5. Bioaerosol Sampling

For the collection of viable virus from aerosol, a liquid impactor/tangential nozzles impinger—namely the BioSampler (SKC Inc., Eighty Four, PA, USA)—connected to the end of the cylindrical aerosol transmission tube—has been used for collecting the bioaerosol in 20 mL of infection medium. The sampling duration was 5 or 10 min. The sampling flow was 12.5 L per minute guaranteed by a TCR Tecora Bio Bravo pump. The pump was protected from virus contamination by an impinger containing NaClO as a disinfection agent, followed by a dryer filled by silica that treats the air sucked. At the end of the procedure, the liquid sample was collected in a falcon tube and the SKC container was washed in distilled water and refilled with a new medium.

### 2.6. Viral RNA Load after Aerosolization

SARS-CoV-2 viral load was measured in the liquid collected in the SKC biosampler, in the BLAM nebulizer after bio-aerosolization, and in the initial virus suspension.

Briefly, 15 μL of the liquid were mixed with 45 μL of distilled water and submitted to thermolysis for 3′ at 98 °C, followed by 5′ at 4 °C.

Then, SARS-CoV-2 was quantified by Real Time PCR using for target the N (nucleocapsid) gene (CDC primers and probe: 500 nM forward primer GGG AGC CTT GAA TAC ACC AAA A, 500 nM reverse primer TGT AGC ACG ATT GCA GCA TTG, 125 nM probe FAM-AYC ACA TTG GCA CCC GCA ATC CTG-BHQ1, Eurofins, Luxembourg and Luna Universal Probe One-Step RT-qPCR Kit; New England Biolabs, Ipswich, MA, USA) on the 7500 Fast Real-Time PCR instrument (Thermo Fisher Scientific, Waltham, MA, USA, protocol: 50 °C for 10′, 95 °C for 1′, and then 40 cycles at 95°for 10″, 60° for 30″). The nCoV-CDC-Control Plasmid (Eurofins) was used to produce the standard curve for the quantification. The N gene was selected based on previous environmental sampling recovering infectious particles from air samples [40,41,42].

### 2.7. Infectivity Assessment

Infectivity of the collected bio-aerosol was assessed through its inoculation on the Vero E6 cells.

Vero E6 cells were maintained in MEM + 10% fetal bovine serum, 2 mM glutamine, and 100 U/mL penicillin/streptomycin (Euroclone, Pero, Italy). The day prior to the experiment, 200,000 cells were seeded on 6 multiwell plates.

Briefly, after bioaerosolization and sampling, the collection liquid was filtered through a 0.2-μm filter to remove bacteria and debris eventually present in the apparatus that could possibly interfere with the analysis. The supernatants of the Vero E6 cells were removed, and the 20 mL of the bio-aerosol collected was divided in two wells of 6 multi wells plate.

The liquid present in the nebulizer after the aerosolization (about 3 mL) was also tested for the infectivity since the collection procedure may have an impact on the virus viability; the initial dilution of the virus (about 1 mL) was used as positive controls of virus replication.

The cytopathic effect (CPE), characterized by cellular rounding, vacuolization, and detachment, was monitored at the EVOS XL Core Cell Imaging System (Thermo Fisher Scientific, Waltham, MA, USA) for 7 days.

At the end of the period, 15 μL of the supernatants were harvested, mixed with 45 μL of water and virus quantification was performed as described above.

### 2.8. Statistical Analysis

The Mann–Whitney test (KW) non-parametric test was used to compare the viral load between day 0 and day 7 for each experimental condition. The R statistical software [54] was employed for the analysis. The experiments were performed in 4 replicates in 2 independent days.

## 3. Results

Our experimental set-up allowed us to generate SARS-CoV-2 bioaerosol and to collect infectious virus after bioaerosolization.

The first analysis conducted, the determination of the viral load after aerosolization, showed that it was possible to transport a high virus titer through the 30-cm travel chamber. With all the dilutions (10^5^, 10^4^, 10^3^ PFU/mL) and the aerosolization timing (5′ and 10′) tested, RNA signals in the liquid collected in the bio-sampler were detected (Table 1).

**Figure 2:**
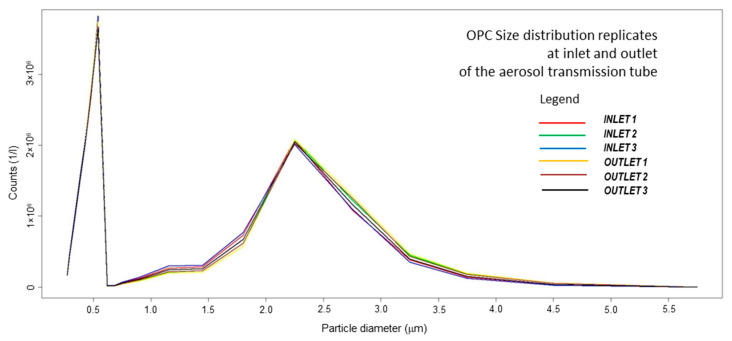
Size distribution replicates determined by Optical Particle Counter, reporting average number concentrations (particle counts/liter) for each of the 20 dimensional bin spanning the range from 0.25 to 5.0 μm (particle diameter of the center of the bin, in micrometers). Three replicate cycles (16 size spectral acquisition each) are measured at the inlet (3 runs) and three cycles were acquired at the outlet of the aerosol transmission tube.

The size distribution of the aerosolized infection medium generated by the 8-Jet BLAM nebulizer operated as Multi Pass Atomizer has been assessed with three independent measurement cycles both at the inlet and at the outlet of the aerosol transmission tube. The size spectrum measured by optical particle counter Grimm EDC 107 ranges from 0.25 to 5.0 μm and it is constituted by 20 dimensional bins. One size spectrum is acquired every 6 s. Reported measurement cycles are each formed by 16 size spectra, each reporting particle counts for each of 20 dimensional bin, acquired in 6 s. The results appear highly reproducible, with no substantial change in number and size of the of aerosol particles within the tube, showing a bimodal distribution with the first mode at 0.5 μm and concentration of 3.7 million of particles/liter and the second mode at 2 μm and concentration of 2 million of particles/liter. Small particles can coagulate, generating bigger droplets that, in the considered settings, stay well smaller than 5 μm. Average concentrations (particle counts/liter vs. particle diameter in micrometers) of the aerosolization runs (15 measures each) measured at the inlet (3 runs) and idem at the outlet (3 runs) of the aerosol transmission tube are reported in Figure 2. Boxplots for size distributions during aerosolization are reported in Supplementary Figure S4. During aerosolizations, the average RH has been 77% (min/max 65/90%) and temperature 20.5 °C (min/max 19/21) in the flow.

The aerosol generated by the considered nebulization system does not belong to the course/droplet mode, but has a distribution with modes smaller than 5 μm, thus fully locating the present research among the SARS-CoV-2 aerosol transmission studies [43,44,45].

Comparing the RNA viral load between the nebulization chamber residues at 0 day post inoculation (dpi) and the initial dilution, a minimal lack of virus was determined. Instead, confronting the RNA quantity between the nebulizer and the bio-sampler, a reduction of 2 Log was found for all dilutions tested.

The second analysis of RNA viral load quantification was set at the 7th dpi. This time point was set in order to allow the viral replication for a sufficient period, also when low quantities of virus are present [55] and to avoid cellular senescence and detachment.

At 7th dpi, an increment of the viral load was observed for the initial 10^5^ and 10^4^ PFU/mL conditions. For the nebulizer and the initial virus dilution, the increase was evident for the three dilutions with a microscopically visible CPE, characterized by cellular vacuolization, rounding, and detachment (Supplementary Figure S5). For the samples collected after the 30-cm tube, although a higher yield was observed by using the molecular test for all the concentrations, there were visible CPEs at 7th dpi only for the dilution 10^5^ PFU/mL for both the 5′ and 10′ of sampling. The statistical analysis showed that the increment of viral load was statistically significant for the dilution at 10^5^ and 10^4^ PFU/mL (Mann–Whitney MW test, 10′ sampling, 10^5^ PFU/mL *p*-value = 0.0001; 5′ sampling, 10^5^ PFU/mL *p*-value = 0.001; 10′ sampling, 10^4^ PFU/mL *p*-value = 0.02; 5′ sampling, 10^4^ PFU/m *p*-value = 0.02) with a increment of ~2–3 Log for the initial 10^5^ PFU/mL and 10^4^ PFU/mL; meanwhile, for the 10^3^ PFU/mL, no differences were observed. It has been observed that 2–3 Logs could be considered as the cut-off to discriminate between replication and no replication in vitro [55,56]. For the dilution of 10^5^ PFU/mL (~2.5 Logs of increment) combined with CPE, the viral amplification was undoubtable; meanwhile, for the other dilutions, the ~2.5 Logs of increment measured was in the range, but the CPEs were not distinguishable, thus the viral replication cannot be declared with absolute certainty, but it can only be supposed.

In Table 1, the average viral load of the experimental conditions (expressed as RNA viral copies for ml), the CPE presence and the results from the statistical analysis are reported.

## 4. Discussion

With our set-up, consisting of BLAM aerosol generator, a travel chamber, and a SKC biosampler for aerosol collection, SARS-CoV-2 was efficiently transported along the 30 cm of the cylindrical tube.

The here proposed experimental model brings different amounts of infective virus in aerosol phase. It approximates a transmission from an infected individual having (1) very high viral load (10^5^ PFU/mL, in the order of 10^7^ RNA viral copies/mL) as viral loads modelled by Shijven et al. [57] and experimental evidence reported by Pan et al. [19]; (2) viral loads considered by [57] in simulations for risk assessment (10^4^ PFU/mL, in the order of 10^6^ RNA viral copies/mL), and (3) lighter viral load from less critical phase of disease (10^3^ PFU/mL, 10^5^ RNA viral copies/mL). The number of particles emitted by the BLAM aerosol generator in the order of million particles is compatible with single sneezing or repeated cough acts. The size distribution with modes at 0.5 and 2 μm at the end of the aerosol chamber/cylindrical tube excludes droplets with diameters of 5 μm or more, having low environmental persistence, due to gravitational deposition. The sampling flow by the SKC Biosampler (12.5 L per minute) mimics inhalation during moderate activity, and sampling periods of 5 and 10 min represent short contacts between infected and susceptible individuals. Dilution in viral loads from the aerosol generator can also emulate environmental dilution in the very short range, and thus the increase of distance between infected and susceptible individuals.

Prior to our work, few studies investigated the SARS-CoV-2 behavior in aerosol in laboratory-controlled settings, mainly in tests aiming at assessing the viability persistence in air. Van Doremalen et al. aerosolized SARS-CoV-2 and SARS-CoV-1 by employing a three-jet Collison nebulizer, Goldberg drum, and gelatin filters for sampling. They found a 3-h stability of both viruses with a slight reduction of viable viruses in aerosol particles <5 µm [44]. Fears et al. sampled replication competent SARS-CoV-2 (diameter particle = 1–3 µm) with an impinger device until 16 h from aerosolization, showing normal morphology on the SEM images [45]. Smither et al. [43] aerosolized a SARS-CoV-2 variant (SARS-CoV-2 England-2 isolated from a 23-year-old male collected in January 2020, GSAID Accession ID EPI_ISL_407073) in tissue culture medium and artificial saliva, by using a 3-jet collision nebulizer, a Goldberg drum, and a midget impinger for aerosol sampling. They found a viable virus until 90 min in both mediums and with medium (40–60%) or high (68–88%) RH; employing artificial saliva, the recovery titer was minor, although with high RH (68–88%), the theoretical decay rate was slower respect to tissue culture medium. Approximately, a reduction of 4 Logs of virus titer (measured as Median Tissue Culture Infectious Dose TCID50) was determined after aerosolization, a little more than our results for the high viral load, although the virus quantification is different.

With the molecular determination of the viral copies, we are able to precisely define the amount of virus (viable and not viable) that can travel across the system. Indeed, by using the 8-jet BLAM nebulizer and the SKC Biosampler, we were able to recover a high quantity of viral RNA for all the dilutions tested.

After 7 days, an increment of RNA viral load (of about 2–3 Logs) was highlighted for the 10^5^ and 10^4^ PFU/mL tested conditions. Interestingly, only the virus derived from the initial 10^5^ PFU/mL was still able to produce visible CPE, even with 7 days of virus infection, indicating the collection of replicative competent virus and a productive infection in the VeroE6 cells.

A statistically significant increment of the viral load was also observed with the 10^4^ PFU/mL, although at microscopic level no effect was determined. Finally, with the more diluted sample (10^3^ PFU/mL), no increment in the level of SARS-CoV-2 was measured and no CPE was found. A possible bias in the quantification can be due to the fact that the entire medium collected during the experiments was maintained in contact with the cells during all the 7 days, without the classical absorption of 1 h with 100–200 µL of volume, followed by the supplementation with new fresh medium. Therefore, during quantification, both viable and not viable viruses were measured. Moreover, the presence of subgenomic viral RNA (sgRNA) should be considered. sgRNA are produced during the viral cycle and are composed by a sg Leader sequence, a transcriptional regulatory sequence, the target subgenomic gene, and the rest of the 3′ of the gene. The N gene is transcribed in sgRNA prior to translation, and it could be a marker of virus amplification, although in diagnostic samples, it is not always an indicator of active replication [58]. However, this procedure was selected in order to avoid all the possible loss of viable virus due to the dilution in the 20 mL of volume medium used in the SKC biosampler and to be sure to detect all the possible variations in the viral load. Therefore, taking into consideration these technical issues, the combination of molecular test and the observation of CPE became fundamental to definitely assess the infectivity.

With this study, we were able to define the minimal quantity of SARS-CoV-2 required (10^5^ PFU/mL) to establish a productive infection in vitro after an aerosolization procedure. The confirmation of productive infections of the samples collected is mandatory, since the merely viral RNA quantification is not a valid indicator of the efficiency of the customized system.

We are aware that the SARS-CoV-2 genomic RNA quantification of virus stock employed in the analysis overpasses of several log the infective titer (genomic copies to PFU ratio ~10^4^), but this can be expected since it is known that the infective fraction of SARS-CoV-2 is less in respect to the genomic quantification; indeed, it was previously measured a genomic copies to PFU ratio of 10^5^–10^6^ [59]. Of note, we evaluated the virus quantity of the stock at the 3rd dpi, while Klimstra et al. did so at the 4th dpi; thus, the different evaluation of genomic copies to PFU ratio can be expected [59].

An interesting study by Ratnesar-Shumate compared the efficiency of eight different aerosol sampling devices in recovering SARS-CoV-2 nebulized in artificial saliva. The SARS-CoV-2 was loaded in the nebulizer at 10^5.7^ TCID50/mL, with a size distribution mode of the aerosolized particle of 1.6 µm. The maximal concentration of virus recovery was 10^3.5^ TCID50/L of air with the Biosampler, while the less performant devices were glass and perfluoroalkoxy fluoropolymer midget impingers. The other five, namely, all-glass impinger, Sioutas impactor, BC 251 sampler, gelatin, and polytetrafluoroethylene filters loaded on Delrin filter holders resulted in the same yield of viral load. They measured the viral presence during 3 min of aerosolization and then continued the sampling for 30 min without a significant loss, apart from when the impingers were tested [60]. These results are a confirmation of superior performance of the SKC biosampler used also in the present work.

Notably, the previous studies analyzed only a single titer of virus, 10^5.25^ TCID_50_ per milliliter (20–22 cycle threshold) [44], ~10^1^ PFU/L aerosol/~10^4^ viral genome/L aerosol [45], 10^6^ TCID_50_/mL). Considering the conversion TCID_50_/_mL_ = 0.7 × PFUs/mL [61], we can assume that, in our study, the viral concentrations used initially in the nebulizer were quite similar and comparable to those in the former and current studies.

## 5. Conclusions

Despite the fact that the direct airborne transmission of SARS-CoV-2 is a matter of debate in the scientific community, the aerosol mode of transmission is unquestionable [62]. The study of SARS-CoV-2 in aerosol in controlled laboratory conditions can be useful to determine its viability, but also to define the minimal dose able to establish a persistent infection as we assessed in our study.

By employing highly efficient of 8-jet BLAM nebulizer and biosampler, SARS-CoV-2 RNA was recovered after a short (30 cm) aerosol transfer, although replication competent virus was observed only when the initial viral concentration was 10^5^ PFU/mL. The proposed experimental setup for flow transmission of viable viruses has been tested on SARS-CoV-2 and it shows the feasibility of testing viral filtration efficiency also on this viral strain; the bio-aerosol measuring train has a potential for testing disinfection and bioaerosol sampling technologies operating at low flow. The effects on the residual infectivity of airborne transmitted virus of the variation of environmental parameters as temperature, relative humidity, atmospheric particulate matter, atmospheric oxidants, and solar UV rays have not been considered here, and studies on these factors represent research needs for a satisfactory elucidation of the fate of SARS-CoV-2 in real scenarios. Research is planned on these lines, as a follow-up of the experimental steps described in this study.

The reported experimental results contribute to support the evidence of aerosol transmission of SARS-CoV-2, able to replicate on cell cultures, even after a very short time (5 min) of aerosolization, with a particle size well below 5 μm.

These results contribute to strengthen the experimental basis for the consideration of this virus as an airborne pathogen and, bearing in mind this mode of transmission, for addressing mitigation and preventive measures that should be undertaken, with a special attention to the monitoring of indoor air quality and virus contamination.

## Figures and Tables

**Table 1:** Viral load (average viral copies for ml) at day 0 and at day 7. The data for 5 and 10 min of sampling, from the residual liquid in the nebulizer, and for the initial dilution prior to testing are reported. Cytopathic effects (CPE) and *p*-value from the results of Mann–Whitney test (MW) are also shown. ns = not significant statistical analysis.

	10^5^ PFU/mL				10^4^ PFU/mL				10^3^ PFU/mL			
	Day 0	Day 7	CPE	MW	Day 0	Day 7	CPE	MW	Day 0	Day 7	CPE	MW
10′ sampling	9.1 × 10^7^	6.2 × 10^9^	+	0.0001	4.6 × 10^6^	1.2 × 10^9^	-	0.02	9.9 × 10^5^	9.7 × 10^5^	-	ns
5′ sampling	4.2 × 10^7^	3.7 × 10^9^	+	0.001	2.4 × 10^6^	2.3 × 10^8^	-	0.02	5.4 × 10^5^	9.9 × 10^5^	-	ns
nebulizer after aerosolization	2.1 × 10^9^	1.6 × 10^10^	+	0.03	4.1 × 10^8^	1.4 × 10^10^	+	0.03	3.4 × 10^7^	8.2 × 10^9^	+	0.03
Initial prior to testing	5.1 × 10^9^	9.1 × 10^9^	+	ns	4.2 × 10^8^	1.3 × 10^10^	+	ns	4.6 × 10^7^	5.4 × 10^9^	+	ns

## Data Availability

Data are contained within the article or Supplementary Material.

## Supplementary Materials

The following are available online at https://www.mdpi.com/article/10.3390/ijerph182111172/s1; Supplementary File S1, virus strain information; Figure S1: Scheme of the bio-aerosol sampling train; Figure S2: BLAM nebulizer; Figure S3: SKC BioSampler; Figure S4: box plot of particle counts; Figure S5, representative images of cytopathic effect in Vero E6 cells.
