# Supplementary material for: Evaluation of Residual Infectivity after SARS-CoV-2 Aerosol Transmission in a Controlled Laboratory Setting
Source: Int J Environ Res Public Health. 2021 Oct 24;18(21):11172. doi: 10.3390/ijerph182111172 (PMC8582643; doi:10.3390/ijerph182111172)
Supplement: Supplementary file 1 [file ijerph-18-11172-s001.zip › ijerph-1397232-supplementary.pdf]

Supplementary information

Virus strain

The SARS-CoV-2 strain is a clinical isolate previously employed in other published studies (Zupin et al. Journal of Biophotonics, 2021, https://doi.org/10.1002/jbio.202000496; Zupin et al. International Journal of Environmental Research and Public Health, 2021, https://doi.org/10.3390/ijerph18179020).

The sample was initially tested with the diagnostic assay Allplex SARS- CoV-2 assay, Seegene, Seoul, South Korea on the CFX connect Real Time PCR detection system, BioRad, Hercules, CA, USA, used for routine clinical analysis.

Then it was tested for the major circulant variants by employing the SARS-COV-2 VARIANTS REALTIME PCR KITS (detecting B.1.1.7; B.1.351, B.1.1.28.1 (P.1), B.1.525 strains; Vircell Microbiologist, Granada, Spain), and the REALQUALITY SARS-CoV-2 AIM Variants (detecting AY.1/AY.2, B.1.617.2/B.1.617.1/B.1.617.3, B.351/B.351.2/B.351.3 strains, AB analitica, Padua Italy).

The sample was negative for the major variants investigated.

Supplementary Figure S1

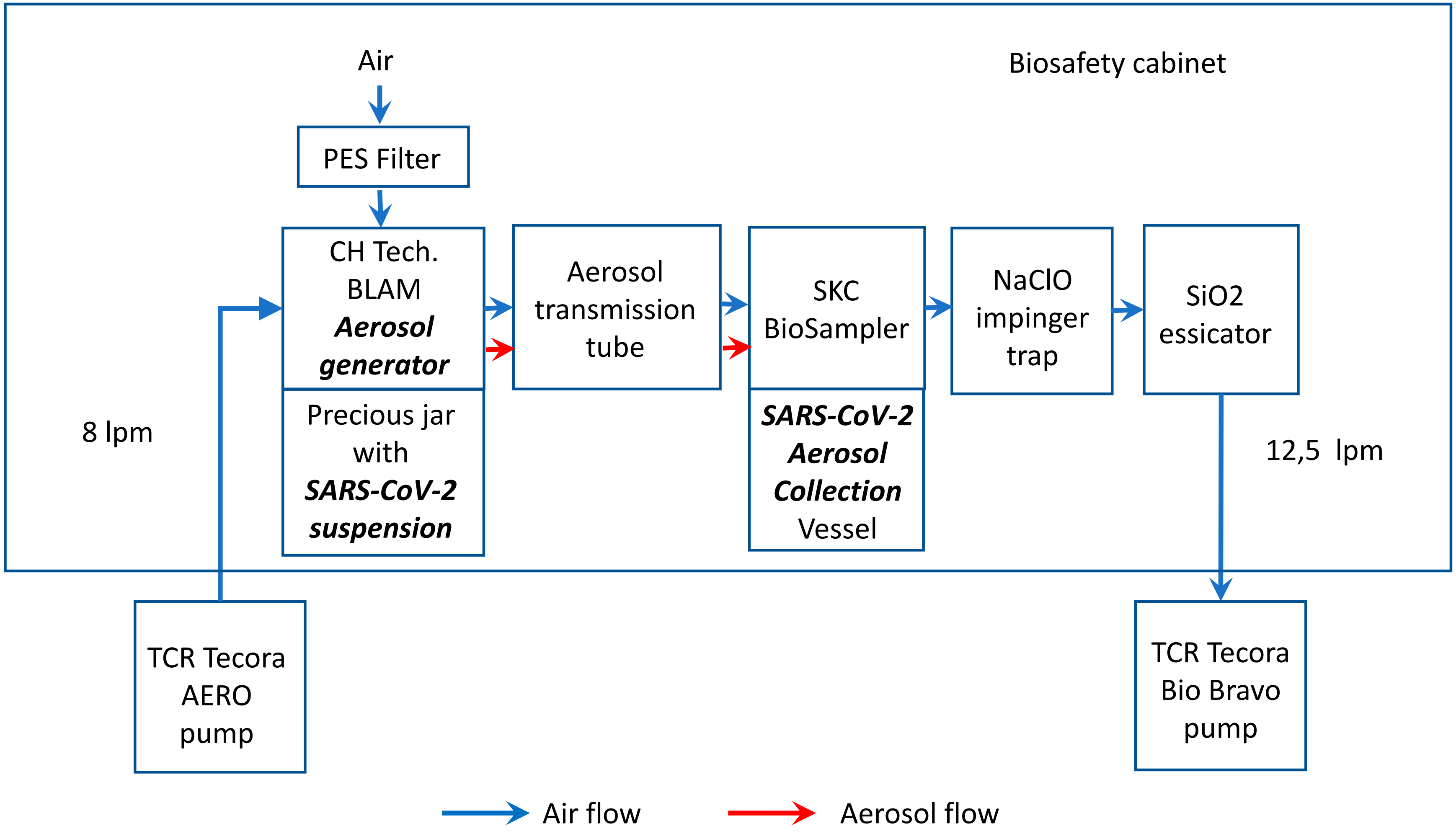

Supplementary Figure S1: Scheme of the bio-aerosol sampling train showing the air flow (blue arrow) and aerosol flow (red arrow).

TCR Tecora Aero pump generate a 8 liter for minute flux in the BLAM aerosol generator containing SARS-CoV-2 suspension. The aerosol is transferred in the aerosol transmission tube and then actively collected by the SKC Biosampler. A TCR Tecora Bio Bravo pump is used to aspirate the flow at 12.5 liter for minute. NaClO impinger trap and SiO2 essicator are employed to inhibit the virus carry over to the pump, while the PES filter is used to clean the incoming air and to avoid accidental exit of virus.

Supplementary Figure S2

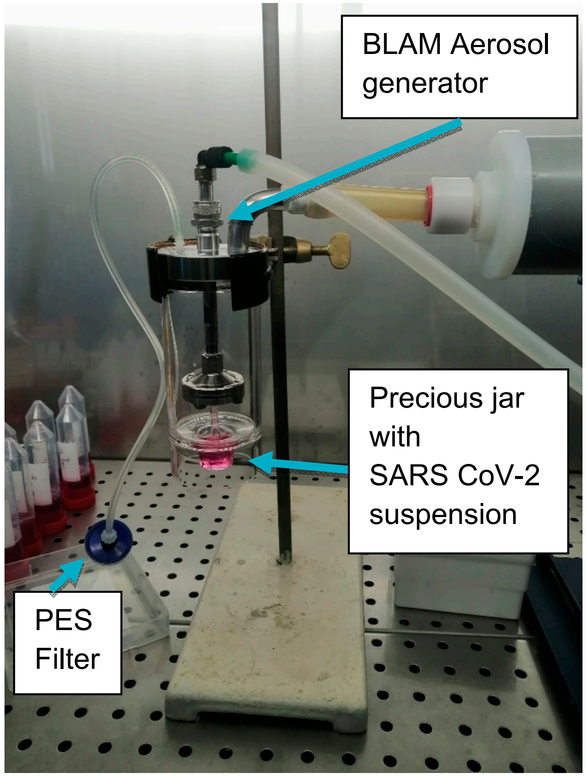

Supplementary Figure S2 : BLAM Aerosol generator with SARS-CoV-2 suspension (pink color)

Supplementary Figure S3

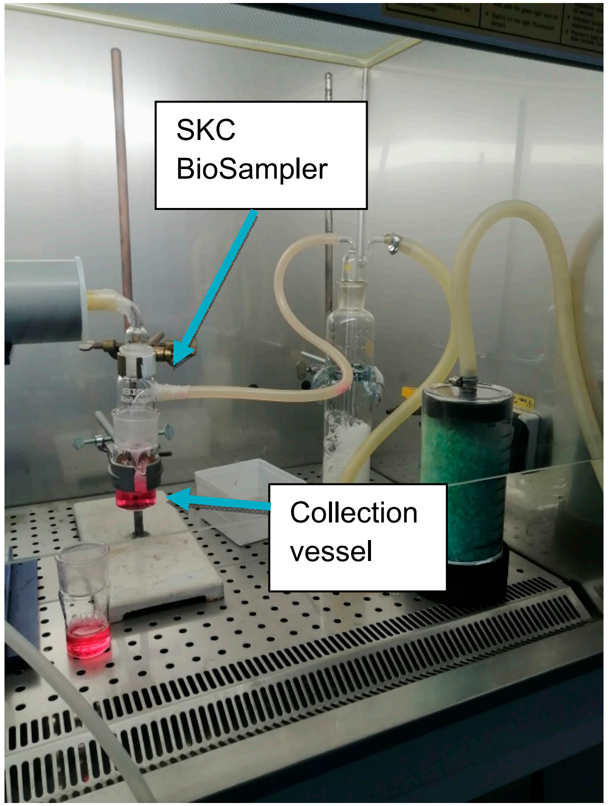

Supplementary Figure S3 : SKC BioSampler with collection vessel (pink color)

Supplementary Figure S4

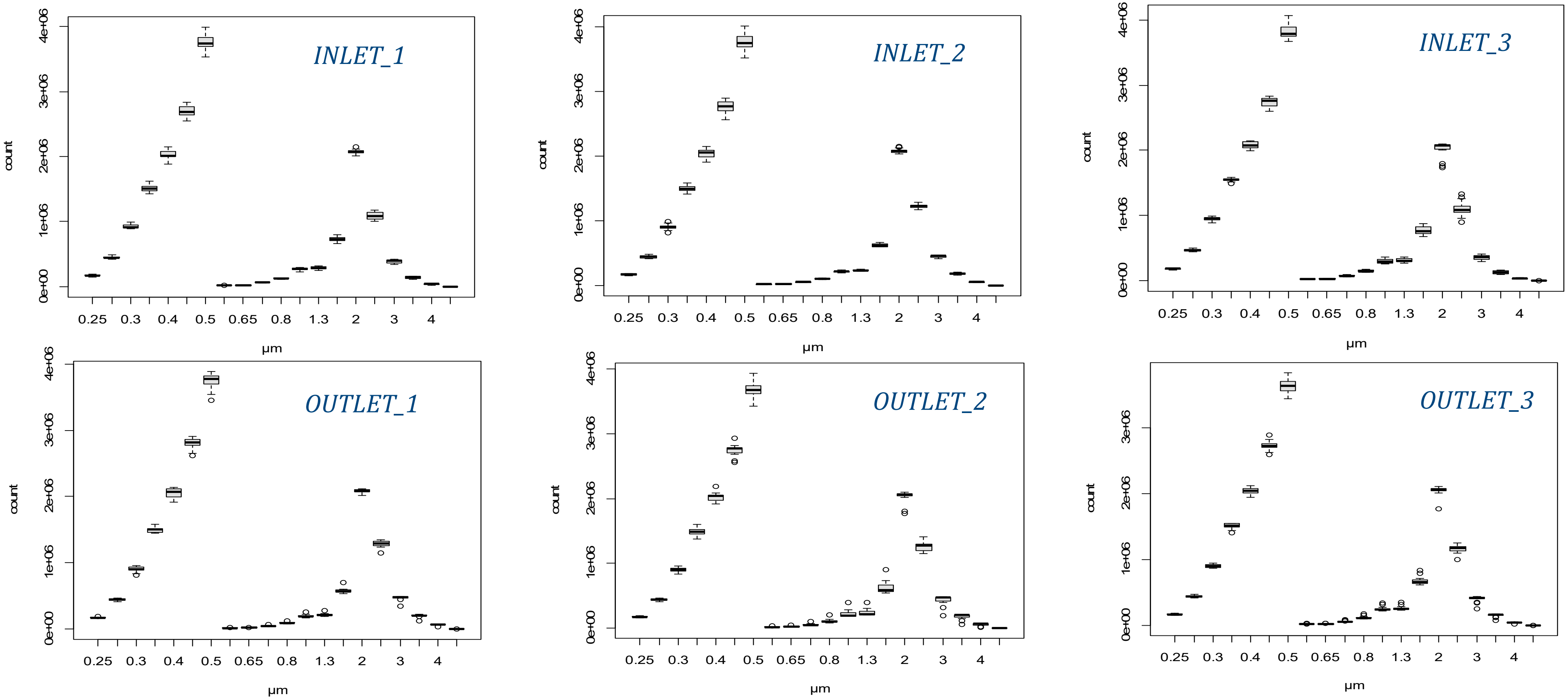

Boxplots for particle counts at size bins from 0.25 to 5 microns from aerosolization cycles measured by optical particle counter Grimm EDC 107 for aerosol from infective medium (MEM + 2% fetal bovine serum, 2 mM glutamine, and 100 U/ml penicillin/streptomycin), generated by 8 jet BLAM in multiple pass atomization mode. INLET_1 to _3 refers to three measurement cycles with sampling with T junction positioned between BLAM nebulizer and aerosol transmission tube from the line of bioaerosol measuring train shown in Figure 1 from the paper; OUTLET_1 to _3 refers to three measurement cycles with sampling with T junction from the line of bioaerosol measuring train positioned after the aerosol transmission tube. Each measurement cycle has 16 spectral size counts of 6 seconds (i.e. each single boxplot has 16 points, covering 96 seconds of aerosolization).

Supplementary Figure S5

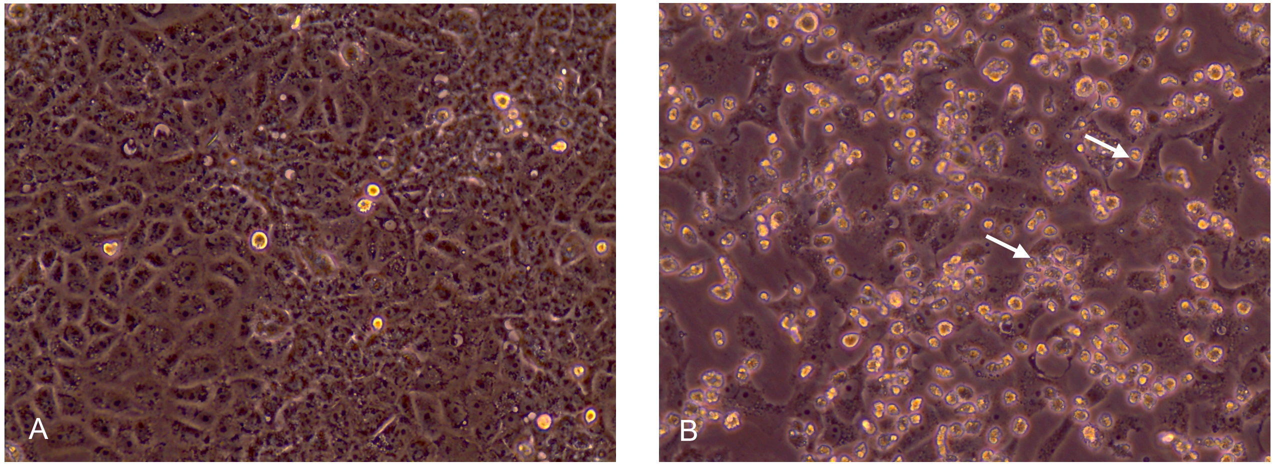

Representative images of Vero E6 cells at the 7th days post infection,

A not treated cells

B cytopathic effects induced by SARS-CoV-2: rounding cells, vacuolization, abnormal stressed shaping, cellular detachments are visible (white arrows).
